# The Antivivisection Bill

**Published:** 1908-03

**Authors:** 


**A Monthly Review of Medicine and Surgery.**

*EDITOR:*

WILLIAM WARREN POTTER, M. D.

All communications, whether of a literary or business nature, books for review and exchanges, should be addressed to the editor 238 Delaware Ave., Buffalo, N. Y.

Vol. LXIII. MARCH, 1908. No. 8

### The Antivivisection Bill

ANTIVIVISECTION is becoming a fashionable fad; at least, many fashionable people have joined the ranks of the opponents of physiologic experiments upon animals. Some doctors even have stooped to indorse the antivivisection movement and have been quoted thus in order to lend charactei to the attack on scientific progress.

A dangerous bill has been presented to the legislature which, under cover of regulating the practice of vivisection, would strike a heavy blow at animal experimentation as carried on in the laboratories.

The Medical Society of the State of New York has appointed a committee on experimental medicine which held a meeting in New York, January 15, 1908, and passed resolutions relating to this subject which are printed elsewhere. It is well understood by most physicians that existing laws relating to the prevention of cruelty to animals adequately safeguard experiments to prevent abuses.

In the issue of this Journal for February we made editorial reference to the fact that a petition was being circulated among physicians indorsing the bill referred to. At this time we would further accentuate what we said on that occasion, and also invite attention to an extract from an editorial in the *Brooklyn Daily Eagle* for January 14, 1908, which presents the subject with great intelligence and fairness. Vice-chancellor McKelway, inter ala, wrote:

The medical profession of the state of New York is a civilised body of educated and humane men. The different schools of medical education and practice report candidates for admission to medicine and surgery to the examining board designated by the regents, and that examining board insists on like qualifications for all intending practitioners. Neither those already in practice, nor those hereafter to become practitioners require any such overseership as the proposed bill suggests, and they do not need to make any medical state official a clearing house, so to speak, to pass upon their vivisectional surgery or their use of anesthetics or any other department of their art.

The *Buffalo Express* in its edition of February 6, 1908, coldly comments on the situation in this manner:

The council of the Medical Society of the County of Erie takes a positive stand in asking physicians and surgeons to withhold their signatures from a petition in favor of a legislative bill "to prevent cruelty by regulating experiments on living animals." The strongest conceivable argument against vivisection is inherent in every human soul. Normal minds recoil from the simple proposition of torturing animals. Yet science has a cold and logical answer for the merciful: It is in the interests of humanity that vivisection is practised at all. It is to be noted, however, that the bill protested against does not absolutely prohibit the experiments, but prevents "cruelty by regulating experiments." A mass-meeting to crystallise and estimate sentiment against vivisection is to be held in New York on February 14th, and some strong and condemning talk may be expected.

It had been our belief, heretofore, that the intelligence of the *Express* would prevent it from falling into the error committed by so many laymen, that lower animals are tortured during experiments made in the interests of science in the laboratories.

It is refreshing in this relation to quote the remarks made by Mrs. Cadwalader Jones, president of the woman's auxiliary of the Society for the Prevention of Cruelty to Animals, at a meeting held at the headquarters of the society February 18, 1908.

Mrs. Jones was careful to explain at the outset that what she was about to say was only her personal opinion and did not in any way represent the auxiliary, which took no stand upon a subject regarding which humane persons were divided. Mrs. Jones began by saying that she had been a lover of animals from her childhood. She had even petted many of the lower orders, and for thirty-five years had never slept without a dog of some kind on her bed. Nevertheless, she believed that vivisection was absolutely necessary. She then went on to say:

"Twenty years ago I saw children in the hospitals choking to death in the agonies of diphtheria," she said. "I saw my only brother die slowly in agony. And no one who has ever seen such things can regret the experiments upon animals that have given us antitoxin. Formerly lacerated wounds of the intestines, made by gunshot and other means, were nearly always fatal.

Now surgeons have learned by experiments on dogs how to operate in such cases. Diseases that could not be studied on animals cost the lives of many thousands of women before physicians learned how best to operate. Now the mortality is small. Vivisection is necessary for the training of physicians. Would you trust yourself to a chauffeur who had never seen a machine in operation?

No amount of dissection after the heart is stilled in death will enable the student to understand the operation of the valves. But valves may be studied in a calf's heart while the animal is under the influence of ether, and it can be killed before it becomes conscious, suffering much less than the animals that are killed for food."

Mrs. Jones added that she had no doubt many cruel experiments were made upon animals, and urged the members if they heard of any such to follow them up, but she was sure they must be comparatively few, since it would take "four men to hold a dog or cat if it were operated upon without anesthetics."

After reading so much dilettanteism displayed by men in discussing this topic, it is refreshing to note that a woman can approach the subject with fairness and present it with forceful and convincing argument. Let us hope the legislature will be influenced by Mrs. Jones and the friends of her inclining.

---

WINTER sports are all very well when properly conducted, but promiscuous snowballing on the streets of a populous city should be prohibited. Many accidents have already been reported in this city and probably many others have escaped the notice of the authorities.

As a result of a letter sent to Superintendent Emerson, of the education department by Chief of Police Regan, a lecture was read in many of the public schools recently, cautioning the pupils against throwing snowballs. The boys were told to confine their snowballing to their companions, and not to pack the snow. Chief Regan cited numerous accidents that have resulted from snowballing.

We have called attention heretofore to the disorderly conduct of pupils on the streets after dismissal from school and this lawlessness has increased since snowy weather began.

---

ONLY two months ago we published, under Topics of Public Interest, a statement by Dr. A. H. Doty, health officer of the port of New York, that no danger lurked in soiled banknotes as a medium of infection. Now comes a press dispatch under date of February 19, 1908, announcing that John McD. Hopkirk, of New

York, is dead of malignant scarlatina as the result of handling poisoned money. Mr. Hopkirk was cashier of Mills Hotel No. 2, in which cheap lodgings are given to the poor, and in that capacity handled hundreds of dirty bills from the slums of the city.

---

About a month ago a dinner was given at the Buffalo Club at which it was proposed to bring the different institutions of Buffalo of an educational character into coöperation concerning the establishment of a Greater University. Those present were Mayor J. N. Adam, John W. Robinson, president of the Chamber of Commerce; Andrew Langdon, president of the Buffalo Historical Society; Dr. Lee H. Smith, president of the Society of Natural Sciences; Willis O. Chapin, president of the Fine Arts Academy; E. H. Butler, president of the board of trustees of the Grosvenor Library; Thomas T. Ramsdell, president of the Buffalo Public Library: Walter L. Brown, librarian of the Buffalo Public Library; Charles P. Norton, vice-chancellor of the University of Buffalo; R. R. Hefford, Carleton Sprague, William H. Gratwick, John J. McWilliams, the Honorable T. Guilford Smith and H. H. Littell.

It was proposed that the city should purchase a portion of the old Pan-American site which should be added to the park system. Upon this site might be placed the botanical gardens, the new Natural Sciences building, one of the public libraries or a branch of one or both and the University buildings. The idea met with so much favor that Senator Hill was asked to introduce a bill in the legislature authorising the city to bond itself for the purpose of buying the land. Senator Hill has already introduced such a bill which we trust will meet the approval of the legislature.

---

Dr. H. W. Wiley, chief of the bureau of chemistry of the Department of Agriculture, is quoted as saying that the expulsion from the body of such drugs as borax, benzoic acid, benzoate of soda, sulphate of copper, sulphur dioxide, formaldehyde and salicylic acid, when contained in foodstuffs, is performed almost entirely by the kidneys, and that he is satisfied the term of American life would be lengthened if the use of such drugs in foods were wholly discontinued.

In view of the prevalence of kidney disease among Americans it would be well to eliminate every possible source of the cause.

---

The French Academy of Sciences, according to a recent cable dispatch, listened sympathetically to an address by Dr. Savary, of

Paris, who warned his hearers against the dangers of rising immediately upon awakening from sleep. He said the strenuous business-man who bolts out of bed to his bathtub the moment he opens his eyes in the morning renders himself liable to a number of ailments, including madness.

It is absolutely necessary, Dr. Savary declared, to rest wakefully in bed for twenty minutes before getting up.

---

A way of avoiding the headache which usually goes with the consumption of champagne has been found. Many who used to eschew the pleasures of the sparkling wine now drink it without difficulty and without fear of bad results. They have adopted an English custom and drink Apollinaris with the wine. The mineral water contains a little bicarbonate of soda, and this, with its other properties, gives it power to relieve acidity of the stomach.

---

The commission of Portuguese residents of Rio Janeiro, according to a recent dispatch, which has been collecting funds for the reception of King Carlos of Portugal, who was to have come here on a visit, this year, has determined to appropriate this money for the erection of a tuberculosis sanitarium to be called after Queen Amelia. The principal infirmaries of the proposed institution will be named after King Carlos and Prince Luiz Philippe.

